# A systematic review of resprouting in woody plants and potential implications for the management of urban plantings

**DOI:** 10.1002/ece3.10839

**Published:** 2024-01-17

**Authors:** Claire Kenefick, Stephen Livesley, Claire Farrell

**Affiliations:** ^1^ Green Infrastructure Research Group, School of Agriculture, Food and Ecosystem Sciences The University of Melbourne Melbourne Victoria Australia

**Keywords:** coppicing, disturbance, fire response, naturalistic planting, resprouter, urban

## Abstract

Naturalistic plantings, such as meadow‐style plantings, can improve the quality of urban green spaces through aesthetic, biodiversity and low maintenance features. Species selection for, and maintenance of naturalistic plantings are key to their success. While herbaceous and grassy meadows can be mowed, naturalistic plantings with woody plants require more intense maintenance to remove biomass and promote resprouting. We aim to understand woody plant responses to diverse disturbance regimes to potentially inform the selection and management of woody species in urban plantings. We conducted a quantitative systematic literature review of 72 papers and investigated what main external (climate, disturbance regime) and internal (buds, life stage, storage reserves) factors influence the resprouting response of woody plants. We found resprouting literature is geographically widespread for woody plants, but studies are skewed towards Temperate climates in USA and Australia, with a focus on high severity and high frequency fire disturbance. Resprouting response was mostly defined as a continuous response to disturbance dependent on disturbance regime, climate and plant traits. Maintenance and management of naturalistic woody plantings, through hard pruning techniques such as coppicing, may be informed by analogous high severity and high frequency disturbance studies. However, the literature on woody plant resprouting has several knowledge gaps for lower severity and lower frequency disturbance regimes and in more arid climates. Future research should evaluate the response of naturalistic woody plantings to disturbance in specific urban contexts.

## Graphical abstract

We systematically reviewed 72 papers on woody plant resprouting. We assessed the literature to understand how resprouting is researched with disturbance regime, climate, plant life stage, meristematic bud availability and carbohydrate storage reserves. The results of this review have the potential to inform the design and management of urban woody plantings.

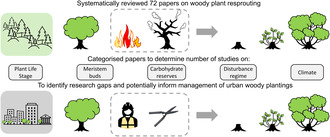

## 1 INTRODUCTION

Many urban plantings are typically either high maintenance annual flower beds, or low maintenance, simplistic and homogeneous grass swards with little visual appeal (Hitchmough, 2000; Norton et al., 2019). An alternative is the ‘naturalistic meadow planting’, which can be low maintenance but achieve high biodiversity, aesthetic and habitat values (Alizadeh & Hitchmough, 2020; Bretzel et al., 2009; Hitchmough & Dunnett, 2004). The resilience of meadow plantings depends on how well species are matched with site conditions (Hitchmough & Dunnett, 2004; Norton et al., 2019). In Temperate climates, meadow plantings are generally diverse mixtures of flowering herbaceous plants that are dormant in winter (Alizadeh & Hitchmough, 2020; Hitchmough & Dunnett, 2004). However, in climates with drier summers, diverse plantings of woody flowering shrubs may provide year‐long aesthetic value and may offer an alternative style of naturalistic planting. Increasingly, naturalistic woody plantings are being implemented in urban areas as a low maintenance alternative, particularly at sites with challenging conditions (City of Melbourne, 2020; Farrell & Rayner, 2020). However, the success of these woody plantings will rely on careful plant selection and management regimes to encourage flowering and regeneration.

Shrubland ecosystems are common in climates with hot‐dry summers and cool‐wet winters (i.e. Mediterranean‐type climate) with shrub‐dominated vegetation communities such as the Chaparral, Fynbos, Kwongan, Maquis and Matorral occurring across the globe (James, 1984). Perennial shrubs found in these areas flower prolifically, are visually appealing and may be better adapted to the common growth conditions of many urban landscapes in hotter and drier climates (i.e. low nutrient soils, water limitation; Enright et al., 2012). These shrubland ecosystems are also managed by fire, a common disturbance that many shrubs can recover from by resprouting after the loss of above‐ground biomass (Bond & van Wilgen, 1996a; James, 1984). Fire disturbance promotes diversity in shrubland plant communities by creating gaps via plant death or top‐kill of above‐ground shoots (Bond & van Wilgen, 1996b). Fire also promotes dense regrowth resulting in multi‐stemmed plants with lots of flowers (Midgley, 1996). However, managing naturalistic plantings in highly urbanised landscapes with fire would be hazardous and expensive. Therefore, we need to identify a maintenance technique that removes most of the above‐ground shoot mimicking disturbance by fire and promoting resprouting.

In naturalistic herbaceous meadows, plants are managed through the low maintenance method of mowing that removes above‐ground biomass (Dunnett, 2004). However, a more robust method is required for woody plants. Coppicing is a silvicultural technique where plant stems are removed close to ground level, stimulating resprouting from meristematic buds at the base or from roots of the plant (Ford‐Robertson, 1971). In ecological research, coppicing (also referred to as ‘clipping’) is used experimentally to simulate disturbance regimes such as herbivory, fire and wind throw and is considered an anthropogenic analogue for these naturally occurring disturbances (e.g. Borzak et al., 2016; Fornara & Du Toit, 2008; Hmielowski et al., 2014; Ickes et al., 2003; Nzunda et al., 2008; Schafer & Just, 2014; Vesk et al., 2004). Managing naturalistic woody plantings with coppicing may also be a low maintenance analogue for the mowing management of herbaceous meadows (Kingsbury, 2004). Coppicing aims to promote woody plants with more stems, denser canopies and more flowers, therefore creating a ‘Woody Meadow style planting’ (Farrell & Rayner, 2020). While coppicing may be a useful tool to manage urban plantings, it is important to understand how woody plants will respond to the disturbance. Understanding what factors influence resprouting response could potentially inform plant selection and the disturbance management regime for successful and resilient naturalistic urban woody plantings.

Broadly, resprouting in plants is defined as the vegetative response to disturbance (Clarke et al., 2013). Woody plant resprouting response to disturbance has been defined in two ways, binary or continuous (Bond & Midgley, 2001). A binary framework defines the resprouting response as a discrete reaction to disturbance, generally either a plant dies or resprouts and survives. However, the arbitrary thresholds for plant survival and resprouting can change between studies, making comparisons difficult (Vesk et al., 2004). A continuous framework defines resprouting response as a scaled weak‐to‐strong response to disturbance and this response can change within an individual or species depending on the disturbance severity and frequency (Bellingham & Sparrow, 2000; Bond & Midgley, 2001; Vesk & Westoby, 2004). Understanding the distinction between these definitions is important when assessing the woody plant resprouting response literature and the factors that may influence resprouting response.

Previous reviews of woody plant resprouting response have highlighted several external factors such as site conditions (e.g. disturbance regime, climate) and internal factors such as plant traits (e.g. bud availability, life stage, resource storage) as important influences on resprouting (Bond & Midgley, 2001, 2003; Clarke et al., 2013, 2015; Pausas et al., 2016). Theoretically, resprouting as a response to disturbance should be widespread across all ecosystems and climates as it can be recognised as an ancestral trait in woody angiosperms, dependent on the availability of buds and stored resources (Wells, 1969). However, some studies have suggested that climate influences the resprouting response of woody plant species at the community level (Clarke et al., 2013). Climate dictates what disturbances can occur and how severe or frequent they may be.

The disturbance *regime* is the combination of severity, frequency and type. Many types of disturbance can promote a resprouting response in woody plants including natural and anthropogenic disturbances (Bond & Midgley, 2001). Disturbance *severity* is a measure of the plant‐specific perception of a disturbance event, based on the adaptations the plant has evolved for that disturbance (Bellingham & Sparrow, 2000). In contrast, disturbance *intensity* is a quantifiable energy output produced by the disturbance event itself (i.e. energy released per unit area, per unit time; White & Pickett, 1985). In this systematic review, we will use disturbance severity. Disturbance *frequency* is how often the disturbance occurs. The interaction of multiple disturbance regimes is also important to consider as the effects of each disturbance event may compound and deliver a greater impact on a plant and alter its resprouting response (Kaczynski & Cooper, 2015; Marrinan et al., 2005).

**FIGURE 1:**
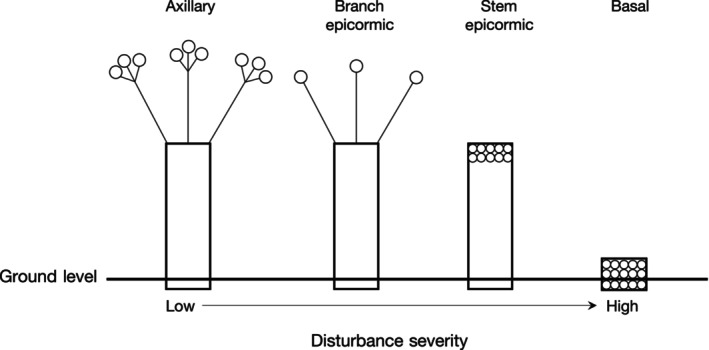
Location of meristematic buds on a plant according to disturbance severity. Circles indicate buds. Adapted from Bellingham and Sparrow (2000).

Clarke et al. (2013) proposed the buds‐protection‐resources framework to understand resprouting in fire‐prone ecosystems. The framework considers if a plant has meristematic buds, protection (height, bark or below‐ground buds) and storage reserves and where each is located in the plant (Clarke et al., 2013). Resprouting can occur from meristematic buds located at different positions on a plant shoot. The buds available for resprouting depend on the disturbance severity and how much of the plant shoot is removed (buds = circles in Figure 1). High severity disturbances that remove all above‐ground biomass and buds will most often result in basal resprouting.

The life stage of a plant can influence its resprouting response as this is often linked to plant height, bud protection and internal resources (Clarke et al., 2013). In a meta‐analysis Vesk (2006) showed broadly that trees lost resprouting ability with age, but shrubs were able to maintain resprouting ability well into maturity. Plants also need adequate storage reserves to resprout after shoots have been removed, this includes starch and non‐structural carbohydrates in roots and stems (Clarke et al., 2013; Nzunda et al., 2008).

Understanding what woody plant resprouting literature is available and where we have knowledge gaps is vital for its potential use to inform urban planting design and management. Therefore, we conducted a systematic and quantitative literature review to assess published academic literature on woody plant resprouting as a response to natural and anthropogenic disturbance. This review focuses on studies that assessed resprouting response in relation to the external factors (site conditions) and internal factors (plant traits) of individuals and species. English language peer‐reviewed academic journals were systematically searched to address the following research questions in relation to woody plant resprouting response:

1. How is resprouting response to disturbance defined?
2. Where has resprouting been studied?
3. Which disturbance types are most researched?
4. What is the most common disturbance regime?
5. What plant traits are studied?

Based on the results of the systematic literature review, we discuss how these aspects of woody plant resprouting response can potentially inform the design of naturalistic ‘woody meadow’ plantings managed by disturbance such as coppicing.

## 2 METHODS

A systematic and quantitative literature review was conducted based on the methods outlined in Pickering et al. (2015) and Pickering and Byrne (2014).

### 2.1 Search procedures

Original research papers published in English language academic journals were sourced from Web of Science and SCOPUS electronic databases (February 2022). Primary TOPIC keywords used in the database searches in Boolean logic were: (Woody plant*) AND (Resprout* OR Sprout*) AND (disturbance). Next, we added one of the following secondary TOPIC keywords for each disturbance type: (fire), (herbivory), (drought), (flood*), (hurricane OR typhoon OR cyclone), (wind), (uproot*), (frost), (snow break OR avalanche), (landslide*). See Appendix S2 for the exact number of papers found for each search term.

As there were several reviews of woody plant resprouting that discuss the definitions, mechanisms and other factors that influence resprouting (Bond & Midgley, 2001, 2003; Clarke et al., 2013, 2015; Pausas et al., 2016), we used reference lists from these reviews and the papers obtained in the online search to find additional primary‐source academic papers. The papers sourced from citation lists are indicated with a note in the literature review database (Appendix S1).

### 2.2 Screening and eligibility

Review papers, opinion papers, meta‐analyses, ‘grey’ literature (e.g. books, opinion pieces) and duplicate papers were excluded from the systematic review. Studies that featured both the primary and secondary TOPIC keywords in the abstract were initially selected. Inclusion criteria were developed to filter abstracts and full texts to select the most relevant papers.

Inclusion criteria:

- Papers must mention a natural or manipulative disturbance.
- Resprouting must be observed or measured as a response to the disturbance event.
- Papers must only consider resprouting in woody plants—studies on other growth forms (e.g. grasses, forbs) were excluded.
- Papers must assess the resprouting response at the plant or species level; studies considering demography change or abundance/proportion of resprouters were excluded as the focus of this review was to determine factors that influence resprouting response of individual plants or species.

Table 1 outlines the screening process for inclusion of literature in this systematic review.

**TABLE 1:** Steps taken in quantitative systematic literature review.

Step	*N*	Notes	Revised *N*
Search	442	Raw combined papers from WoS and SCOPUS	–
Other sources	42	Papers from reference lists of review papers and original papers found in search	484
Duplicates	−225	Removed all duplicate papers from search	259
Non‐original	−22	Removed reviews, opinions, meta‐analyses, books and reports	237
Screening—not relevant	−74	Abstracts that did not fit inclusion criteria were removed	163
Eligibility—not relevant	−91	Full text that did not fit inclusion criteria were removed	↓
Total removed	−412	Papers included in this review	72

Abbreviation: WoS, Web of Science.

### 2.3 Data categories and analysis

For the remaining 72 papers that passed the selection process (Table 1), the following information was recorded in a literature review database (Appendix S1):

1. Basic data on the paper, including author(s), year of publication, paper and journal titles, journal category and keywords.
2. Information on the study type and context, including definitions of resprouting, whether the research was experimental, observational or other (e.g. modelling), and information on the study's geographical location (including coordinates), climate and bioregion (if relevant).
3. Information on the disturbance regime including disturbance type, severity and frequency. Multiple disturbance types or events were collected as distinct occurrences.
4. Information about the studied plant traits, including the location of resprouting on the plant (bud position), life stage and location of carbohydrate storage (if mentioned).

#### 2.3.1 Definitions of resprouting

Studies that defined resprouting as a binary response usually classified plants as either resprouting or dying in response to disturbance. Studies that defined resprouting as continuous used terms such as ‘weak’ or ‘strong’ resprouting response that depended on disturbance severity as in Bond and Midgley (2001).

#### 2.3.2 Disturbance regimes (type, severity, frequency)

The disturbance regime was rarely explicitly stated in the studies included in this review. We needed to investigate the methods, site history and sometimes introduction sections to understand the severity, frequency and type of each disturbance that was reported.

We split disturbance types into *natural disturbances*:

- wildfire (naturally occurring fire, non‐anthropogenic origin),
- drought,
- herbivory (grazing),
- frost,
- wind or hurricane damage (wind throw, storm),
- avalanche (landslides, uprooting, burial),

and *anthropogenic disturbances*:

- planned fire (experimental treatments, management burns, anthropogenic origin),
- coppicing (clipping, cutting) and
- forest clearing (gap generation).

Note, that wildfire and planned fire were separated as distinct categories as they act in different ways as disturbances. Studies of wildfire were mostly observational and reported burns were patchy. Whereas studies of planned fire were a mix of observational and experimental with explicit treatments and generally more consistent burn patterns.

Classification of disturbance severity and frequency was highly subjective, with study authors using different definitions that were often study‐specific. In this systematic literature review, we classified the severity and frequency of disturbances as:

- high severity—all above‐ground biomass lost or ‘top‐kill’,
- moderate severity—partial (>40%) above‐ground biomass remaining alive or ‘crown‐kill’,
- low severity—damage to leaves, twigs or branches only,
- high frequency—occurs more than once in 10 years or ‘chronic’,
- moderate frequency—occurs every 10–30 years,
- low frequency—occurs less than every 40 years or ‘rare’ and
- single event—a single isolated occurrence either observed or performed for an experiment without any disturbance history reported.

Note that several papers assessed either multiple disturbance events, different levels of disturbance severity and frequency treatments, or evaluated the combined effects of two or more disturbance types (e.g. fire and drought, or fire and herbivory). We report the number of occurrences of each disturbance regime across all papers. Therefore, some papers appear multiple times in the Results (Section 3) and in figures.

#### 2.3.3 Plant traits

We classified bud position into four levels based on the hierarchy of Bellingham and Sparrow (2000) shown in Figure 1:

- basal (including below‐ground buds),
- stem epicormic,
- branch epicormic and
- axillary.

We considered two life stage classes for this review:

- juvenile—any plant described as ‘juvenile’, ‘sapling’, ‘seedling’, ‘immature’ or under certain age thresholds according to species, defined by relevant study authors.
- mature—any plant described as ‘mature’, ‘reproductively mature’ or over certain age thresholds according to species, defined by relevant study authors.

We classified storage reserves into two groups:

- above‐ground (e.g. leaves, stems) and
- below‐ground (e.g. roots, lignotubers).

#### 2.3.4 Data presentation

Descriptive statistics, summary tables and figures were generated in R (R Core Team, 2021) using the package ‘tidyverse’ (Wickham et al., 2019) to summarise trends and patterns in the literature and to highlight knowledge gaps. The world climate map was made by reclassifying the 31 Koppen–Geiger climate zones into six broad climate groups (Arid, Continental, Mediterranean‐type, Polar, Temperate and Tropical) and using raster files from the Koppen–Geiger climate classification (Kottek et al., 2006) and the ‘raster’ (Hijmans, 2022) and ‘sf’ (Pebesma, 2018) packages in R. See Appendix S3 for the grouping of Koppen–Geiger climate zones.

## 3 RESULTS

A total of 72 original research journal papers were identified from the systematic search and screening of woody plant resprouting response after disturbance. Most papers were published in Plant Science, Ecology, Evolution and Behaviour & Systematics journal categories (41 papers, 57%; Appendix S2). Within the 72 papers, we recorded 132 occurrences of disturbance.

### 3.1 Definitions of resprouting response

**FIGURE 2:**
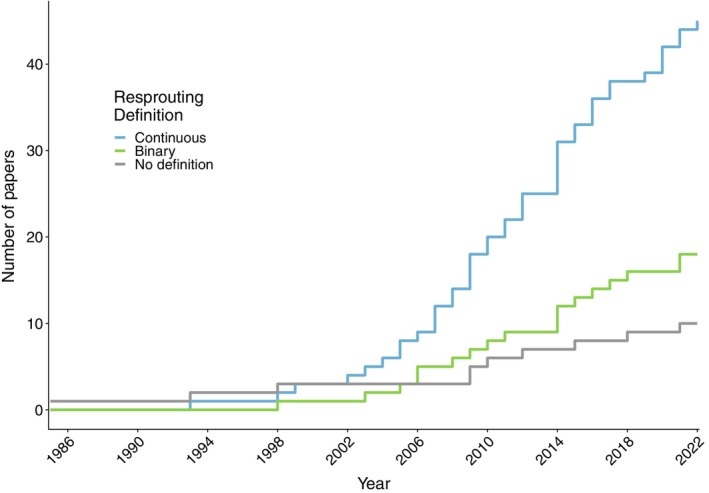
Number of papers reporting each resprouting definition over time.

In this systematic literature review, 63 papers defined resprouting. Most papers used the continuous definition of resprouting (45 papers) over the binary definition (18 papers; Figure 2). The binary response was more common in fire disturbance studies (19 occurrences). Over time, the definition of resprouting has increasingly become continuous with the binary definition being used in fewer studies (Figure 2).

### 3.2 Geography of resprouting

**FIGURE 3:**
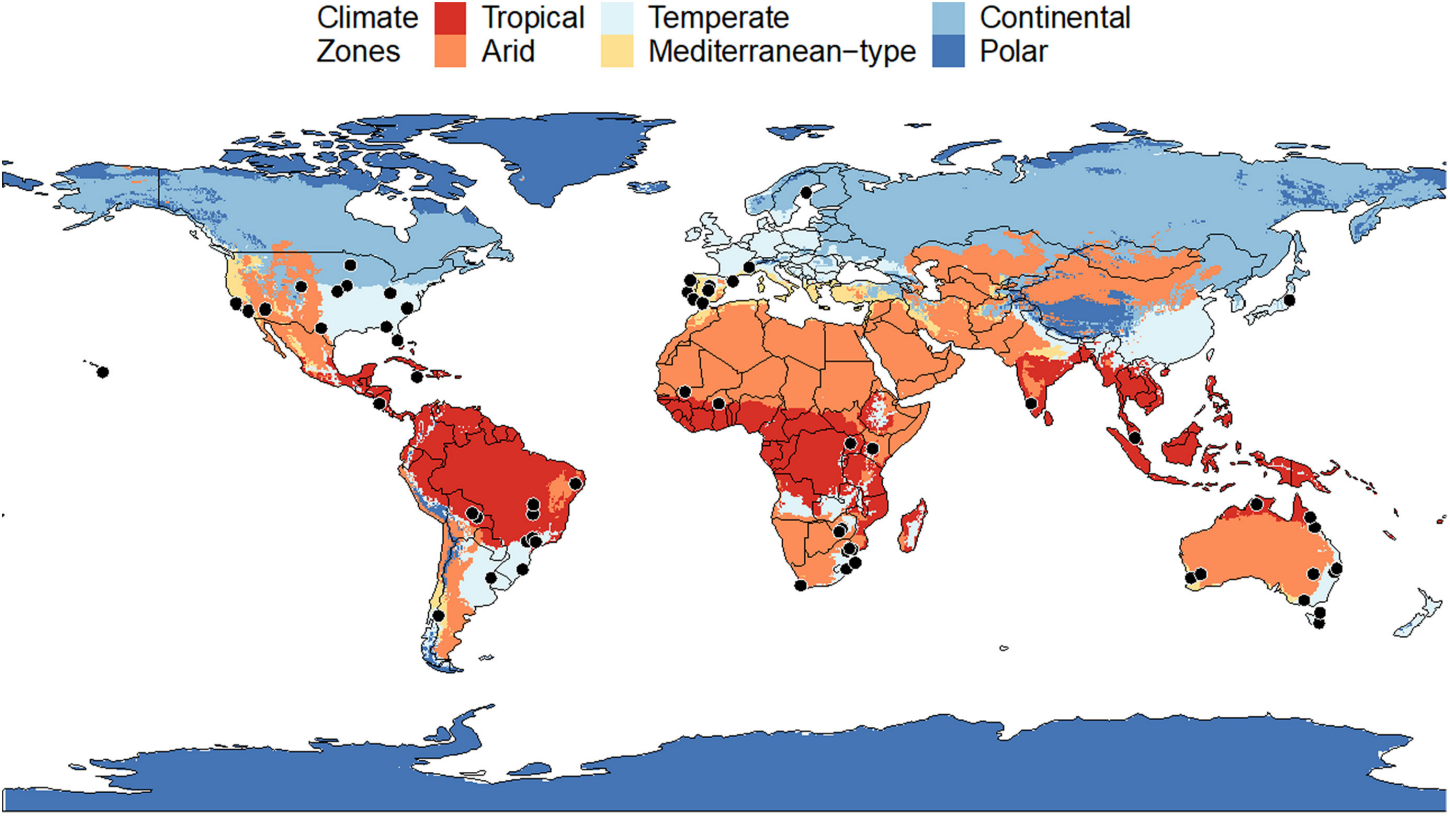
Map showing the 72 study locations (black dots). Colours represent the six climate groups used in this systematic review to evaluate the geographical distribution of studies on resprouting. Climate groups are derived from the Koppen–Geiger world map (Kottek et al., 2006).

Resprouting has been studied in 20 countries spanning both hemispheres (Figure 3). However, most research occurred in the USA with 17 papers (24%), followed closely by Australia with 12 papers (17%). The highest number of studies occurred in Temperate climates (25 papers, 34%) and no studies were carried out in Polar climate zones (Figure 3). The exact number of occurrences in each climate group can be found in Appendix S4.

### 3.3 Disturbance types most researched

**FIGURE 4:**
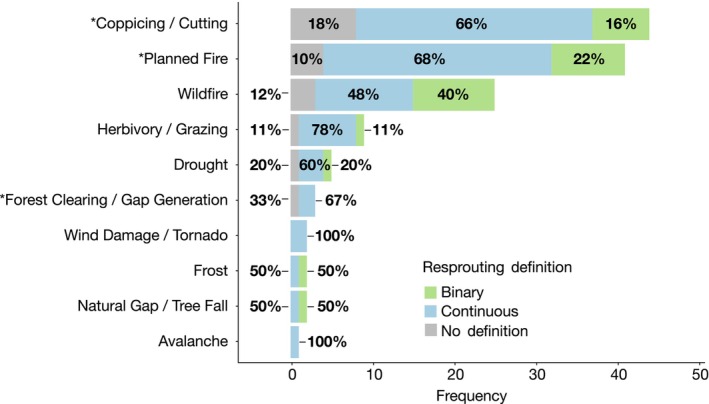
Frequency of each disturbance type in the 72 publications evaluated in this systematic review, split by resprouting definition binary (green), continuous (blue) or no definition (grey). Percentages indicate the frequency of resprouting definition for each disturbance type. *Coppicing/Cutting, Planned Fire and Forest Clearing/Gap Generation are anthropogenic disturbances.

For resprouting in response to natural disturbance, wildfire was the most common type of disturbance with 25 occurrences across the 72 papers (19% of all 132 occurrences). Anthropogenic disturbances also received considerable attention, with 86 occurrences (65%) assessing resprouting in response to coppicing, planned fire and forest clearing combined (Figure 4). The large number of studies evaluating resprouting in response to anthropogenic disturbances is likely due to the experimental nature of these studies (43 papers, 58%), where anthropogenic methods mimicked natural disturbances. Coppicing was the most common anthropogenic disturbance with 43 occurrences (33%), and it was often used as an experimental analogue for fire or herbivory. Planned fire was the next most common anthropogenic disturbance with 40 occurrences (30%).

Studies on woody plant resprouting in response to natural non‐fire disturbances—including herbivory, drought, wind damage, frost, tree fall and avalanche—collectively only made up a fifth of the occurrences (24 occurrences, 18%) in this systematic literature review (Figure 4).

### 3.4 Disturbance regimes

**FIGURE 5:**
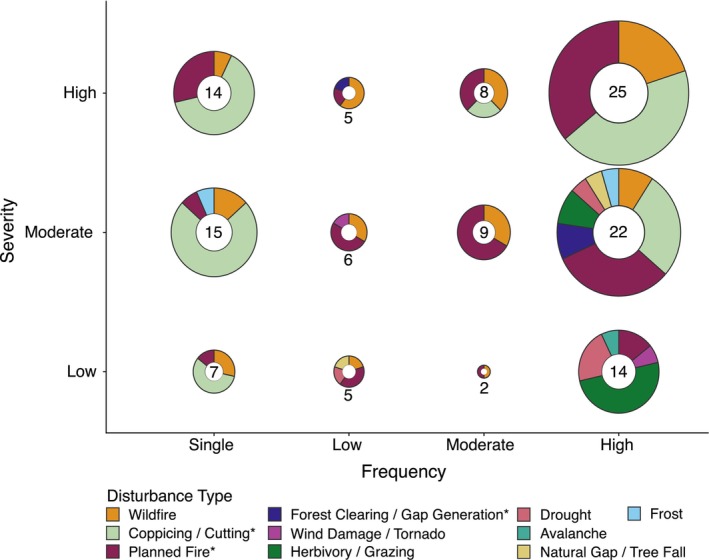
Donut plots representing the severity, frequency and type of disturbance regimes for each occurrence in the 72 papers. The *x*‐axis represents frequency (single event, low [>40 years interval or rare], moderate [10–30 year interval] and high [<10‐year interval or chronic]). The *y*‐axis represents severity (low [damage to twigs or branches], moderate [crown‐kill] and high [top‐kill]). Donut chart size indicates the number of disturbance occurrences in all papers for each severity and frequency interaction (i.e. small radius = a few occurrences, large radius = many occurrences) and the specific numbers are shown in the centre of each donut. Colours within donut charts represent disturbance type, and colour wedge size indicates the number of occurrences for the corresponding colour type in each severity and frequency interaction. *Planned fire, Coppicing/Cutting and Forest Clearing/Gap Generation are anthropogenic disturbances.

Disturbance regimes are a combination of disturbance severity, frequency and type (Figure 5). Resprouting in response to high severity and high frequency disturbance was the most studied regime (25 occurrences, 19%). After high frequency (61 occurrences, 46%), single events were the next most studied frequency with 36 occurrences (27%) across the three severity classes. Far less research occurred with moderate (19 occurrences) and low frequency (16 occurrences) disturbances. Within the high frequency class, moderate severity disturbances had the greatest diversity in the types of disturbances studied (Figure 5). Whereas studies that evaluated high severity disturbances were dominated by fire and coppicing disturbances (Figure 5). The exact number of occurrences in each disturbance regime class can be found in Appendix S5.

### 3.5 Plant traits

There were 67 papers (93%) that assessed and reported the location of resprouting on a plant. Basal resprouting was the most studied location of resprouting (62 papers, 86%), with stem epicormic (23 papers, 32%), branch epicormic (19 papers, 26%) and axillary (4 papers, 6%) resprouting recorded to a lesser extent. Table 2 shows the interaction between the disturbance regime and the position of resprouting on the plant. Resprouting from basal buds was the most common mechanism in studies that assessed high severity and high frequency disturbance regimes (23 occurrences). Whereas stem (eight occurrences) and branch epicormic (nine occurrences) resprouting were recorded mostly in moderate severity but high frequency disturbance regimes. Axillary bud resprouting was absent from all high severity regimes (except for single events) and occurred mostly in moderate and low severity regimes (Table 2).

**TABLE 2:** Interaction between disturbance regime (severity and frequency) and location of resprouting buds.

Severity × Frequency	Basal	Stem epicormic	Branch epicormic	Axillary
High × High	23	3	2	0
High × Moderate	7	3	1	0
High × Low	5	1	0	0
High × Single	14	6	3	1
Moderate × High	18	8	9	2
Moderate × Moderate	7	6	2	0
Moderate × Low	5	3	2	1
Moderate × Single	13	6	4	2
Low × High	10	4	6	1
Low × Moderate	1	2	1	0
Low × Low	5	2	0	1
Low × Single	6	4	4	1
Total	114	48	34	9

*Note*: Each bud position column contains the number of occurrences when resprouting was observed originating at each bud position and may be recorded multiple times in one paper depending on the number of disturbance events.

Mature plants were assessed in 55 papers (62%), juvenile plants were assessed in 33 papers (38%) and 17 papers (24%) assessed both juvenile and mature plants.

In total, 16 papers (22%) considered storage reserves and resprouting. Five papers (7%) considered combined above‐ and below‐ground storage reserves in relation to resprouting. Below‐ground storage reserves (eight papers, 11%) were investigated more than above‐ground storage reserves (three papers, 4%). Of the total 16 papers, all but one used coppicing treatments to investigate the impact of storage reserves on resprouting. This exception assessed below‐ground storage reserves and resprouting after planned fire and drought.

## 4 DISCUSSION

Vegetative resprouting as a response to disturbance has been a focus of plant ecology research for over 100 years (Jepson, 1916), with a particular emphasis on fire disturbance (Busch & Smith, 1993; Gómez Sal et al., 1999; Hodgkinson, 1998; Keeley & Zedler, 1978; Wells, 1969). Resprouting after disturbance is advantageous as it enables plants to regenerate above‐ground biomass in situations where they have successfully established and adapted to site conditions (James, 1984). Historically, resprouting studies have mainly focused on fire‐prone Mediterranean‐type shrubland ecosystems such as the Californian Chaparral (Coffman et al., 2010), South African Fynbos (Marais et al., 2014) and South American Matorral (Raffaele & Veblen, 1998), but there is increasing attention being given to resprouting after fire in Tropical and Temperate Cerrado shrublands (Dodonov et al., 2014). Woody plant species that can resprout have been recorded in open‐access online databases such as the TRY plant trait database (global; Kattge et al., 2020), AusTraits (Australia; Falster et al., 2021), the BROT 2.0 (Mediterranean Europe; Tavşanoğlu & Pausas, 2018) and the USDA PLANTS database (USA; https://plants.usda.gov/home).

Shrublands that are disturbed by fire are diverse vegetation communities tolerant of drought and low nutrient soils (Enright et al., 2012; James, 1984). Due to these features, shrublands have the potential to be used as habitat templates to create resilient naturalistic urban woody plantings that are managed by disturbance and regenerate through resprouting. However, this requires an understanding of how resprouting in woody plants is affected by both external and internal factors such as disturbance regime, climate and plant traits. We found that resprouting response has been recorded in all disturbance regimes, and at all plant life stages and is studied across many climates and countries.

### 4.1 Definitions of resprouting response

Most papers considered resprouting response as a continuum, especially when resprouting was evaluated in response to non‐fire disturbances and in non‐Mediterranean‐type climates (Borzak et al., 2016; Franklin et al., 2010; Gómez Sal et al., 1999; Ickes et al., 2003; Nzunda et al., 2014). Continuous definitions became more common over time as more types of disturbances and lower severity regimes were studied (Botha et al., 2020; Fornara & Du Toit, 2008; Franklin et al., 2010). The binary definition was commonly found in resprouting studies assessing high severity fires or coppicing, where a plant either dies—non‐resprouter—or survives and recovers vegetatively—resprouter (Bell et al., 1996; Robertson & Hmielowski, 2014; Schafer & Just, 2014). The continuous definition was found more broadly across disturbance regimes of lower severity including more types such as herbivory, wind damage, forest clearing and avalanche. Continuous studies often described resprouting response as a scaled weak‐to‐strong response depending on severity with high severity regimes eliminating many ‘weaker’ species (Hermann et al., 2012; Kennard et al., 2002). Prevailing theories for almost 20 years explain woody plant resprouting response as a continuum that is a function of the disturbance regime (Bond & Midgley, 2001; Vesk & Westoby, 2004). In fact, Bond and Midgley (2001) proposed that the binary definition is actually the extreme ends of a poorly studied continuum and a simple alternative in the absence of information about a system. Understanding the difference in resprouting response definitions is important when considering how we may use the literature to inform the species selection and maintenance of urban woody plantings. For example, a fire‐response study that uses a binary framework may indicate a species will not survive disturbance, but a coppicing study may contradict this with information indicating a weak or moderate response. Therefore, we must be careful when using different disturbance regimes to inform the creation of urban plantings.

### 4.2 Geography of resprouting

We found that resprouting has been studied globally in five climate zones, excluding Polar climates, and that most papers have focused on Temperate climates. Geographically, studies were skewed to the global West, with a particular focus on the US and Europe. Arid and Continental climates were underrepresented in this review, this may be due to the distribution of shrublands and woody vegetation mainly occurring on west‐facing coastlines in Mediterranean‐type climates, and in Temperate areas between 32° and 40° latitudes in both hemispheres (James, 1984). Tropical shrublands are less common but appeared in 16 studies in this review. Though arid shrublands are also less common, these studies are underrepresented with only 10 papers. Continental climates typically do not host shrublands, and the studies that did feature in this review mainly considered tree‐dominated communities (e.g. Shibata et al., 2016; Stokes et al., 2012). Key geographical gaps in woody plant resprouting response are in Asia, Africa and Oceania (except Australia), future studies in these areas will benefit the wider resprouting ecology community. The distribution of woody plant resprouting studies is also linked to the disturbance type, severity and frequency, with some regimes favoured over others.

### 4.3 Disturbance types most researched

We found woody plant resprouting has been studied in response to natural disturbances—wildfire, drought, herbivory, frost, wind or hurricane damage and avalanche—and in response to anthropogenic disturbances—planned fire, coppicing and forest clearing (Figure 4). Fire disturbance has provided a focal point for resprouting research, given its prevalence as both a natural and anthropogenic (planned fires or burns) phenomenon (Pausas et al., 2016). Disturbances that can potentially kill the plant, such as fire, hurricane damage, drought, herbivory and landslides were reported to induce the strongest resprouting response (Bond & Midgley, 2001). Some studies in our systematic review looked at co‐occurring disturbances, such as fire and drought (Marrinan et al., 2005; Parra & Moreno, 2017) or fire and herbivory (LaMalfa et al., 2019; Tredennick et al., 2015). These studies are important as they demonstrate the impact of compounding disturbances, which are likely more common in natural settings than isolated treatments in experiments. Anthropogenic disturbances of cutting, coppicing and planned or experimental fire were the most abundant disturbance types in this review. The high number of anthropogenic occurrences is likely due to the experimental treatments conducted by researchers trying to understand the mechanisms behind resprouting by mimicking wildfire or herbivory (Hayashi & Appezzato‐da‐Glória, 2009; Hermann et al., 2012; Nzunda et al., 2008). Natural non‐fire disturbances were the least studied types in this review. Figure 5 shows that non‐fire disturbances are generally associated with lower severity disturbances. Perhaps the reduced severity of these disturbances is part of the reason these regimes are less studied—their impact on plants is not as extreme as the high severity disturbances. Nonetheless, further research on non‐fire disturbances will lead to a greater understanding of woody plant resprouting, especially as a continuum of response.

### 4.4 Disturbance regimes

As in Bellingham and Sparrow (2000), we found woody plant resprouting literature was skewed towards higher severity and frequency disturbance regimes, commonly with binary definitions of resprouting response. We found that resprouting has been defined as continuous in all regimes and this definition is represented consistently across disturbance regimes. In high severity and frequency disturbance regimes, we found the types of disturbance were reduced to three types—wildfire, planned fire and coppicing. These disturbance regimes also resulted in more basal resprouting (Table 2), which is logical when severe disturbances result in top‐kill or complete removal of the above‐ground biomass (Vesk & Westoby, 2004).

For high frequency regimes, more disturbance types were recorded as disturbance severity decreased, with the most disturbance types occurring at moderate severity. Probabilistically, reducing the severity of disturbance will leave more of the plant intact, which means a greater resprouting response can occur from more disturbance types (Vesk & Westoby, 2004).

At low severity, there was a greater diversity in disturbance types, but both low and moderate frequency regimes were underrepresented in the literature reviewed. The higher proportion of single event and high frequency disturbance regimes in this literature review is likely due to the high number of experimental studies looking at coppicing and fire. The short timeframe imposed on many research projects and logistical constraints on experiments may mean that investigations into lower frequencies are often not possible.

### 4.5 Plant traits

Most papers in our review observed basal resprouting, and this type of resprouting was not limited to high severity regimes, indicating that basal buds may be available whenever a plant may need to use them, not just after severe disturbances (Figure 1; Vesk, 2006). Axillary bud resprouting was the least common type, only occurring after moderate and low severity disturbances, except for one study looking at the recovery of trees after severe wildfire (Moreira et al., 2007). Branch and stem epicormic resprouting were also observed more in moderate and low severity regimes. Resprouting from multiple locations was recorded in 27 papers that either had multiple disturbance events or lower severity disturbances. Basal resprouting is likely to be the desirable trait for plants managed with moderate to severe disturbance.

In our review, we found juvenile plants received less attention than mature plants. Bond and Midgley (2001) highlighted the importance of the persistence niche in mature plants, so this may be why more authors focused on understanding resprouting in mature plants rather than in juveniles. Those who have looked at juvenile plants have suggested that resprouting can be a plastic trait and change with life stage (Botha et al., 2020; Ickes et al., 2003; Shibata et al., 2014; Vesk, 2006). The longevity of plants and their ability to resprout into mature life stages is an important factor to consider when designing urban woody plantings.

Below‐ground storage organs such as roots, tubers and lignotubers were the dominant focus of studies that considered storage reserves and resprouting in our review. Thirteen papers assessed resprouting supported by below‐ground reserves in the last 20 years. Above‐ground storage reserves gained more research interest possibly after being identified as a research gap in Clarke et al. (2013), with six papers published after 2013. Storage reserve presence and size were linked to site conditions and resource availability (Paula & Ojeda, 2009; Shibata et al., 2016; Smith et al., 2018); disturbance regime (Menges et al., 2020; Nzunda et al., 2008); and species (Menges et al., 2020). The importance of storage reserves for resprouting has been discussed previously and storage reserves are considered an essential mechanism for resprouting that needs further experimental research and understanding (Clarke et al., 2013).

### 4.6 Potential implications for naturalistic urban woody plantings

Low input naturalistic urban woody plantings need to have year‐long aesthetic value and be tolerant of low nutrient soils and drought. Shrubland ecosystems are a potential candidate for developing these resilient ‘Woody Meadow’ style plantings. In shrublands, fire disturbance manages plant communities resulting in dense multi‐stemmed plants with many flowers (Midgley, 1996). While prescribed fire is still used as a management tool to reduce fuel loads and the risk of wildfire in some fire‐prone countries—such as Australia, South Africa and the USA (Bardsley et al., 2015; Miller et al., 2020; van Wilgen et al., 2012)—it is becoming more contested in peri‐urban and urban areas due to concerns around safety and air pollution (Miller et al., 2020). Therefore, other methods of biomass removal that promote resprouting and are also low maintenance such as coppicing may be a better approach (Dunnett, 2008; Kingsbury, 2004). Coppicing can act similar to fire and mowing by removing most of the above‐ground shoots and promoting a resprouting response (Schafer & Just, 2014). We investigated the resprouting literature to identify what factors—both external environmental and internal plant traits—are commonly associated with woody plant resprouting.

Resprouting in woody plants has been observed in response to natural and anthropogenic disturbances under diverse disturbance regimes. Quantitative research has been conducted across several climate zones and different ecosystems, with useful information on resprouting response coming from Temperate, Tropical, Mediterranean‐type and Arid zones (Busch & Smith, 1993; Chong et al., 2007; Reyes et al., 2009; Sugden et al., 1985; Vanderlei et al., 2021). Low maintenance design and management of urban plantings can be informed by the continuum of resprouting responses. Fire‐response trait databases (e.g. TRY, AusTraits and BROT) could be used to guide species selection for urban plantings, with a preference towards obligate resprouters (binary definition) or strong resprouters (continuous definition). However, care must be taken when using the fire‐response literature for different types of disturbance, especially less severe types (Pausas et al., 2016). Plant responses to disturbance management (e.g. coppicing) may differ due to differences in climate, bud and resource availability, and the interaction of other disturbances post‐maintenance (Poorter et al., 2010). Plants may experience drought or herbivory of new shoots soon after coppicing, so understanding the compounding effects of multiple disturbance events is significant. If coppicing is too frequent or too severe, it could lead to plant death due to insufficient meristematic buds or storage reserves for resprouting (Moreira et al., 2009; Moyo et al., 2015; Paula & Ojeda, 2006; Pelc et al., 2011). Knowledge of the bud position and storage reserves of plants can be used to alter the frequency and severity of coppicing to find the optimal management strategy for naturalistic urban woody plantings. Therefore, information found in woody plant resprouting literature has the potential to create resilient urban plantings through careful species selection and preparation of coppicing management regimes.

## 5 CONCLUSION

We found that resprouting of woody plants is studied globally in almost all climates, except Polar climates. However, the majority of resprouting studies occur in Temperate climates in response to high severity and high frequency fire disturbances. Key factors that influence the resprouting response of woody plants include external factors of local climate and disturbance regime, and plant traits such as bud availability, life stage and storage reserves. Current research focuses heavily on resprouting in response to fire and future research would be strengthened by investigating resprouting in response to non‐fire disturbances, and to low severity disturbances. Greater research into woody plant resprouting in an urban context is essential for designing and managing resilient and diverse naturalistic woody plantings in our towns and cities. With correct inputs, these naturalistic woody plantings could achieve the goal of low cost and low maintenance urban greening for a more sustainable urban future.

## AUTHOR CONTRIBUTIONS

**Claire Kenefick:** Data curation (lead); formal analysis (lead); investigation (lead); validation (equal); visualization (lead); writing – original draft (lead); writing – review and editing (equal). **Stephen Livesley:** Conceptualization (equal); supervision (equal); validation (equal); writing – review and editing (equal). **Claire Farrell:** Conceptualization (equal); resources (lead); supervision (equal); validation (equal); writing – review and editing (equal).

## FUNDING INFORMATION

This research was supported by the Australian Research Council (ARC) Linkage Partnership grant (LP190100536—Resilient and adaptable urban landscapes: low input woody meadows; C.F.), the University of Melbourne Dean of Science Scholarship (C.K.) and Dr Betty Elliott Horticulture Scholarship (C.K.).

## CONFLICT OF INTEREST STATEMENT

There are no conflicts of interest from any of the authors.

## Supporting information


Appendix S1.
Appendix S2.
Appendix S3.
Appendix S4.
Appendix S5.

## Data Availability

The data that supports the findings of this study are available in the supplementary material of this article.
