# Supplementary material for: A systematic review of resprouting in woody plants and potential implications for the management of urban plantings
Source: Ecol Evol. 2024 Jan 17;14(1):e10839. doi: 10.1002/ece3.10839 (PMC10794092; doi:10.1002/ece3.10839)
Supplement: Supplementary file 1 — Appendix S1. Appendix S2. Appendix S3. Appendix S4. Appendix S5. [file ECE3-14-e10839-s001.zip › ece310839-sup-0002-AppendixS2-S5.docx]

Supporting information to the paper

Kenefick, C. et al. A systematic review of resprouting in woody plants and potential implications for the management of urban plantings. *Ecology and Evolution.*

## **Appendix S1.** Database of papers used in the systematic review

See database excel file titled: “Appendix_S1_Final_Paper_Database_2023.xlsx”

Supporting information to the paper

Kenefick, C. et al. A systematic review of resprouting in woody plants and potential implications for the management of urban plantings. *Ecology and Evolution.*

Appendix S2. Number of original research papers examining resprouting response to disturbance per SCOPUS journal classification *category.*

| Journal Category | Number of publications (%) |
| --- | --- |
| Plant Science | 21 (29.2) |
| Ecology, Evolution, Behaviour and Systematics | 20 (27.8) |
| Forestry | 15 (20.8) |
| Ecology | 8 (11.1) |
| Nature and Landscape Conservation | 3 (4.2) |
| General Agricultural and Biological Sciences | 2 (2.8) |
| Global and Planetary Change | 1 (1.4) |
| Management, Monitoring, Policy and Law | 1 (1.4) |
| N/A | 1 (1.4) |
| Total | 72 (100) |

Supporting information to the paper

Kenefick, C. et al. A systematic review of resprouting in woody plants and potential implications for the management of urban plantings. *Ecology and Evolution.*

Appendix S3. Classification of Koppen-Geiger climate zone codes into 6 discrete zone groups.

| Climate Zone Groups | Koppen-Geiger climate zone code |
| --- | --- |
| Tropical | Af, Am, As, Aw |
| Arid | BSh, BSk, BWh, BWk |
| Temperate | Cfa, Cfb, Cfc, Cwa, Cwb, Cwc |
| Mediterranean | Csa, Csb, Csc |
| Continental | Dfa, Dfb, Dfc, Dfd, Dsa, Dsb, Dsc, Dsd, Dwa, Dwb, Dwc, Dwd |
| Polar | EF, ET |

Supporting information to the paper

Kenefick, C. et al. A systematic review of resprouting in woody plants and potential implications for the management of urban plantings. *Ecology and Evolution.*

**Appendix S4.** Number of papers (N) in each climate group. Note that some papers had multiple climate zones studied, so the total is 74 records in of 72 papers.

| **Climate Group** | **N** |
| --- | --- |
| Temperate | 25 |
| Tropical | 16 |
| Mediterranean-type | 15 |
| Arid | 10 |
| Continental | 8 |
| Polar | 0 |
| **Total** | **74** |

Supporting information to the paper

Kenefick, C. et al. A systematic review of resprouting in woody plants and potential implications for the management of urban plantings. *Ecology and Evolution.*

Appendix S5. Number of papers in each severity and frequency combination from Figure 2.

|  | | Frequency | | |  |  |
| --- | --- | --- | --- | --- | --- | --- |
| Severity | Single | | Low | Moderate | | High |
| High | 14 | | 5 | 8 | | 25 |
| Moderate | 15 | | 6 | 9 | | 22 |
| Low | 7 | | 5 | 2 | | 14 |
